# Mobile Health App and Web Platform (eDOL) for Medical Follow-Up of Patients With Chronic Pain: Cohort Study Involving the French eDOL National Cohort After 1 Year

**DOI:** 10.2196/54579

**Published:** 2024-06-12

**Authors:** Noémie Delage, Nathalie Cantagrel, Sandrine Soriot-Thomas, Marie Frost, Rodrigue Deleens, Patrick Ginies, Alain Eschalier, Alice Corteval, Alicia Laveyssière, Jules Phalip, Célian Bertin, Bruno Pereira, Chouki Chenaf, Bastien Doreau, Nicolas Authier, ePAIN, Nicolas Kerckhove

**Affiliations:** 1 Centre d'évaluation et de Traitement de la douleur CHU Clermont-Ferrand Clermont-Ferrand France; 2 Centre d'évaluation et de Traitement de la douleur CHU Toulouse Toulouse France; 3 Centre de Recherche Clinique CHU Amiens Picardie Amiens France; 4 Centre d'évaluation et de Traitement de la douleur CHU Grenoble Grenoble France; 5 Centre d'évaluation et de Traitement de la douleur CHU Rouen Rouen France; 6 Centre d'évaluation et de Traitement de la douleur CHU Montpellier Montpellier France; 7 Analgesia Institute Clermont-Ferrand France; 8 Service de pharmacologie médicale CHU Clermont-Ferrand Clermont-Ferrand France; 9 Direction de la recherche clinique et de l'innovation CHU Clermont-Ferrand Clermont-Ferrand France; 10 Laboratoire d'Informatique, de Modélisation et d'Optimisation des Systèmes Université Clermont Auvergne Clermont-Ferrand France; 11 See Acknowledgments France

**Keywords:** mHealth, mobile health, eHealth, self-monitoring, chronic pain, observational study

## Abstract

**Background:**

Chronic pain affects approximately 30% of the general population, severely degrades quality of life and professional life, and leads to additional health care costs. Moreover, the medical follow-up of patients with chronic pain remains complex and provides only fragmentary data on painful daily experiences. This situation makes the management of patients with chronic pain less than optimal and may partly explain the lack of effectiveness of current therapies. Real-life monitoring of subjective and objective markers of chronic pain using mobile health (mHealth) programs could better characterize patients, chronic pain, pain medications, and daily impact to help medical management.

**Objective:**

This cohort study aimed to assess the ability of our mHealth tool (eDOL) to collect extensive real-life medical data from chronic pain patients after 1 year of use. The data collected in this way would provide new epidemiological and pathophysiological data on chronic pain.

**Methods:**

A French national cohort of patients with chronic pain treated at 18 pain clinics has been established and followed up using mHealth tools. This cohort makes it possible to collect the determinants and repercussions of chronic pain and their evolutions in a real-life context, taking into account all environmental events likely to influence chronic pain. The patients were asked to complete several questionnaires, body schemes, and weekly meters, and were able to interact with a chatbot and use educational modules on chronic pain. Physicians could monitor their patients’ progress in real time via an online platform.

**Results:**

The cohort study included 1427 patients and analyzed 1178 patients. The eDOL tool was able to collect various sociodemographic data; specific data for characterizing pain disorders, including body scheme; data on comorbidities related to chronic pain and its psychological and overall impact on patients’ quality of life; data on drug and nondrug therapeutics and their benefit-to-risk ratio; and medical or treatment history. Among the patients completing weekly meters, 49.4% (497/1007) continued to complete them after 3 months of follow-up, and the proportion stabilized at 39.3% (108/275) after 12 months of follow-up. Overall, despite a fairly high attrition rate over the follow-up period, the eDOL tool collected extensive data. This amount of data will increase over time and provide a significant volume of health data of interest for future research involving the epidemiology, care pathways, trajectories, medical management, sociodemographic characteristics, and other aspects of patients with chronic pain.

**Conclusions:**

This work demonstrates that the mHealth tool eDOL is able to generate a considerable volume of data concerning the determinants and repercussions of chronic pain and their evolutions in a real-life context. The eDOL tool can incorporate numerous parameters to ensure the detailed characterization of patients with chronic pain for future research and pain management.

**Trial Registration:**

ClinicalTrials.gov NCT04880096; https://clinicaltrials.gov/ct2/show/NCT04880096

## Introduction

Chronic pain affects approximately 30% of the general adult population [1,2] and was one of the top 5 leading causes of years lived with disability in 2016 [3], especially among older people [4]. Moreover, 60% of people with chronic pain are less able or unable to work, and 20% report having lost their job as a result [5]. The overall cost of chronic pain is estimated to be approximately €441 billion (US $468 billion) in Europe and US $560 to $635 billion in the United States [6-8]. At the same time, the market for analgesic drugs represented approximately US $68 billion in 2016, and an increase from 2% to 5% was forecast for 2021, with a further 5% increase by 2025 [9]. Unfortunately, available analgesics often have limited efficacy, with undesirable effects and without any real pharmacological innovation [10], despite prolific basic research [11].

One of the solutions could be based on better patient characterization to offer physicians decision aids in the management and initial choice of treatment for chronic pain. A preliminary step that would make it possible to identify patient trajectories and factors capable of predicting treatment success. This characterization should cover the entire biopsychosocial field. It should also include a temporal dimension, with real-life monitoring of different parameters, and subjective and objective markers of chronic pain. Indeed, the current assessment of patients with chronic pain provides only fragmentary data on their daily pain experiences due to memory bias [12]. This monitoring strategy is currently being developed by several research teams [13-18]. It is also necessary to make the patient an active participant in their own care, with the goal of improving compliance and adherence. Indeed, evidence suggests that the self-management of chronic diseases reduces hospitalizations, the use of emergency services, and overall care management [19,20].

The use of innovative digital technologies (eHealth) appears to be a solution. eHealth can improve the characterization and monitoring of pain symptoms, and the management of pain and associated comorbidities [21-23]. eHealth can also offer remote interventions without the difficulties inherent in long-distance travel or regular visits to the general practitioner or specialist [24]. Thus, recent publications have highlighted the urgent need to develop, assess, and use validated eHealth programs for chronic pain [13,14,18,25-31]. eHealth technologies encompass a wide range of diverse tools, offering potential applicability for chronic conditions [32,33]. These technologies can be categorized into 6 types: virtual visits, electronic health records, digital therapeutics, artificial intelligence and machine learning, wearable monitors, and mobile health (mHealth) apps [32]. This last category can vary in its content, which can generally be grouped into 3 objectives: education, monitoring, and treatment (often including self-management strategies), sometimes with a combination of these objectives [34]. However, there are many obstacles in putting this management into practice and monitoring it via digital tools, including the lack of training of patients in the management of their pathology and the use of digital tools, the lack of interest or training of some physicians for this new management integrating new digital technologies, the lack of integration of these digital strategies in medical management, the over-medicalization of pain that can result from the use of these digital tools, and the lack of time and information in health care structures [35].

Change is therefore necessary to integrate digital technology in the management of chronic diseases, including pain [36]. Among digital tools, mHealth offers significant opportunities for patients to foster communication with their practitioners or other patients (ie, through forums). mHealth offers distraction strategies, provides information and therapeutic education, promotes self-expression, improves access to health care, and facilitates social support. Moreover, mHealth can provide patients with greater convenience and more regular access to information about their conditions compared to traditional care. Although many studies have examined the use of innovative mHealth to assist in the management of chronic diseases, less attention has been paid to chronic pain [37]. However, the context is favorable, with growing interest from health agencies and funding organizations for these solutions. Moreover, in France, patient demand was evidenced by the results of a survey on compliance conducted in early 2017 by the French Institute of Public Opinion. The results showed a strong demand by patients for the development of digital tools for the self-management of their chronic diseases.

In response to this situation, the Analgesia Institute (Clermont-Ferrand, France) has recently created an mHealth tool (called eDOL) for data collection and monitoring of chronic pain and its impacts on patient well-being. Following a feasibility study on the use and acceptability of eDOL Version 1 [38] and the implementation of Version 2, we provide in this article an overview of eDOL’s ability to retrieve a significant amount of medical data of interest for physicians and researchers on the subject of chronic pain. This study was carried out after more than a year of existence of the eDOL national cohort, and it included more than 1000 patients. This study did not assess the impact of the eDOL tool on patients’ medical management or on patients’ pain symptoms and their consequences.

## Methods

### Ethical Considerations

The study was approved by the research ethics committee (Comité de Protection des Personnes Ouest II Angers; reference number: 2020-A02027-32) and the French Data Protection Authority (Commission Nationale de l'Informatique et des Libertés [CNIL]; n° 921059). The study has been registered at ClinicalTrials.gov (NCT04880096). The study was conducted in accordance with French laws and regulations on research on human beings and data protection, and in accordance with the Declaration of Helsinki [39].

### Data Collection

Data were collected and managed using the eDOL tool (mobile app + web platform) developed by Bepatient (Paris, France) and hosted by Avenir Télématique (Villeneuve-d'Ascq, France). In accordance with the provisions relating to the confidentiality of information concerning, in particular, the people who took part in the research and the results obtained [40], individuals with direct access took all the necessary precautions to ensure the confidentiality of the information relating to the participants. These persons and the investigators themselves are subject to professional secrecy [41]. All data collected and transmitted to the sponsor (University Hospital of Clermont-Ferrand, France) were anonymized, and each patient had an individual numerical code. The head of research ensured that each patient was informed of which data were collected and that they did not object to their use or disclosure. Answers to questionnaires and medical data were transmitted in spreadsheet format (Excel 2013; Microsoft Corporation). All anonymized data were accessible to the biostatisticians (BD and BP), coordinator (ND), and project manager (NK). Only the investigators could access their patients’ personal data to identify them. A dashboard linking patients’ identities and individual numerical codes was available only on the investigators’ professional interface on the eDOL web platform. The final database, which was used for statistical analyses, included only individual numerical codes to preserve anonymity.

### Data Entry and Processing

To manage data processing, a data warehouse has been set up in the Laboratory of Informatics, Modelling and Optimization of the Systems (LIMOS) Mixed Unit of Research (UMR 6158) (Clermont Auvergne University, France).

This warehouse has made it possible to retrieve the anonymized data collected by BePatient regularly and automatically. The data were retrieved via a secure application programming interface (API). Files were saved and read to extract the data. These data were then cleaned according to a set of rules to remove erroneous data (patient input errors), integrate them into a database, and provide an interface to view and export the cohort data.

### Study Design and Population

This cohort study provides a snapshot after 1 year of a national cohort of patients with chronic pain using the eDOL app. The characterization and real-life monitoring of patients from 18 pain clinics and follow-up by 80 investigators in France took place between September 14, 2021, and January 31, 2023. The study was offered to all physicians and allied health professionals in the investigating centers. Participation in the study was offered to patients with chronic pain, who were owners and regular users of a smartphone and who were followed up in a pain clinic. All adult (≥18 years old) patients able to read and understand French and provide consent to integrate the cohort study were included. Participants were free to withdraw their consent at any time by informing the sponsor. Each patient had access to the information document (paper or electronic) detailing the purpose, content, and conduct of the cohort. If they agreed to participate, they were asked to download the eDOL app and complete questionnaires using the eDOL app. The URL to access this app was sent by email from physicians to their patients. After downloading the app and creating their profiles, patients could accept the general terms and conditions of use and confirm that they agree to the use of their medical data in this study. Each patient made only 1 initial study visit during which the physician introduced the study to them, checked their eligibility, explained the eDOL tool, and gave them a brief training document on how to use the eDOL app. Participants completed several questionnaires and assessments over a period of 1 month (initial patient characterization) and then repeatedly (or not) quarterly, half-yearly, and yearly depending on the questionnaire. Throughout the duration of the study, physicians saw their patients at several follow-up visits (optional), according to the patient’s health pathway, after the inclusion visit.

### eDOL Digital Tool

**Figure 1:**
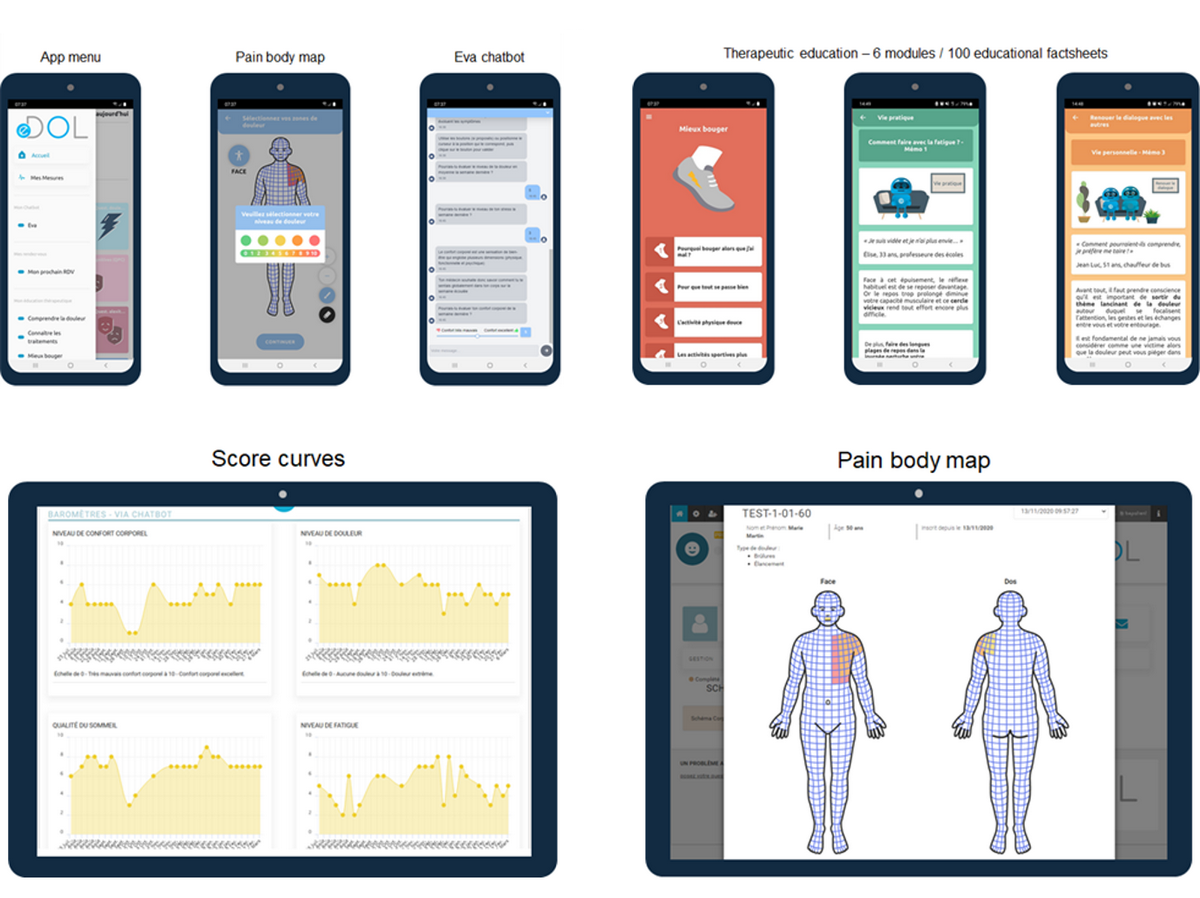
Interface of eDOL Version 2. Screenshots of the various modules of the eDOL smartphone app for patients (top) and the eDOL web interface for clinicians (bottom).

eDOL is a digital health tool used for data collection and monitoring related to pain and its impacts on patient well-being. It comprises 3 main components: a smartphone app for patients, a web interface for health care professionals (Figure 1), and a data repository for researchers. The tool provides various features and modules to support patients in managing their chronic pain while allowing physicians to access and monitor real-time data for clinical and therapeutic purposes.

#### Patient Mobile App

The app allows patients to regularly input data regarding their pain and its effects and to access educational content. Patients were invited to complete various contents, but none of them were compulsory.

#### Questionnaires

Patients complete questionnaires and assessments on a regular basis. These questionnaires cover general aspects, specific chronic pain symptoms, and associated comorbidities. They are filled systematically at different intervals, such as once only, quarterly, half-yearly, or yearly. Some questionnaires address specific types of chronic pain. For a detailed list of all the questionnaires used, refer to the previous publication by Kerckhove et al [38].

#### Weekly Meters

The app facilitates the real-life monitoring of various parameters related to pathology. Patients can assess the evolution of their pain and its impacts using an 11-point numeric rating scale (from 0 to 10) for intensity assessment in different areas, such as pain, stress and anxiety, fatigue, sleep, morale, and bodily comfort.

#### Body Map

Each month, patients can use a body map to select and indicate the painful areas on their body, assigning corresponding intensity levels. The map provides front and back views (Figure 1).

#### Chatbot

Patients can download a chatbot (conversational agent called Eva) to integrate it into the app. The Eva chatbot has been developed in collaboration with the company Aliae (Metz, France) and with a medical group of the Analgesia Institute. The chatbot prompts participants to complete their meters on a weekly basis.

#### Educational Modules

The app offers educational modules that cover therapeutic education on pain and its broader aspects. These modules consist of informative sheets with images for better comprehension. They have been developed with the assistance of a group of physicians working in various pain clinics. Each module starts with a “What you need to know” section, defining the chapter’s scope, followed by educational sheets comprising an image corresponding to the subsection, a short testimonial or quote, an explanatory text, and a personalized image with a concluding statement. Modules include understanding pain, understanding treatments, moving better, improving fatigue and sleep, functioning in daily life, and managing emotions.

In the event of technical problems or questions regarding the app or the completion of questionnaires or meters, participants could refer to contact details provided via an email, and for each investigator center, a clinical research assistant was available by phone.

#### Web Interface for Health Care Professionals

The eDOL web platform enables health care professionals to access real-life patient data (questionnaire scores and graphical presentation of weekly meters) and input consultation data (clinical examinations, treatments, etc). The platform features an ergonomic dashboard with tabs for management (patient medical records), health measures (graphical display of weekly assessments), and questionnaires (all completed with scores and answers).

#### Big Data Repository for Researchers

The development of eDOL V2 involved the creation of a data repository in collaboration with the LIMOS. The data repository interfaces directly with eDOL V2 and automatically collects patient cohort data (via the app) and data from health care professionals (via the web platform) on a weekly basis. The transferred data are anonymized to comply with the French data protection authority for analysis purposes. Information on the data contained in the database is available to investigators on request. In the event of a clinical study requiring eDOL data, a written request describing the study must first be sent to the data controller (Analgesia Institute). The Analgesia Institute’s Scientific Committee will assess the relevance of the project before giving its approval.

### Study Outcomes

The primary objective of this study was to describe the tool’s ability to retrieve a large amount of longitudinal medical data and its relevance to cohort patient follow-up.

The secondary objectives were to assess the characterization of participating patients, pain disorders, pain medications, and related comorbidities, and evaluate the acceptability of the eDOL app with a satisfaction survey (based on the Patient Satisfaction Questionnaire Short Form [42] and the Client Satisfaction Questionnaire [43,44]) for patients (10 questions). The satisfaction survey (in French language) was sent to each included patient via the eDOL tool. Response options for each question ranged from 0 (strongly disagree with the statement) to 10 (strongly agree with the statement). The questionnaire/weekly meter completion rate and center participation (inclusion rate) were also calculated.

As an exploratory study, the profile of patients adhering to the eDOL program was analyzed.

### Statistics

Patients have been described according to the following variables: compliance with eligibility criteria, epidemiological characteristics, and clinical and treatment characteristics. Categorical data are expressed as numbers and associated percentages, and continuous data are expressed as means with standard deviations or medians (25th-75th percentiles), according to the statistical distribution. The assumption of normality was studied using the Shapiro-Wilk test. Comparisons according to observance were conducted using the chi-square test or Fisher’s exact test for binary variables, whereas comparisons concerning quantitative variables were performed using ANOVA or the Kruskal-Wallis test. When appropriate (omnibus *P* value <.05), a post-hoc test for 2×2 multiple comparisons was applied: Tukey-Kramer test after ANOVA or Dunn test after the Kruskal-Wallis test. Different analyses were carried out according to the level of use (compliance) of the eDOL app, which was defined by quartiles of percentage weekly meters completed during follow-up (Q1: <5%, Q2: <50%, and Q3: >50%). Statistical analyses were performed using Stata software (Version 15; StataCorp). All tests were 2-sided, with an α level set at 5% for statistical significance.

## Results

### Generalities

**Figure 2:**
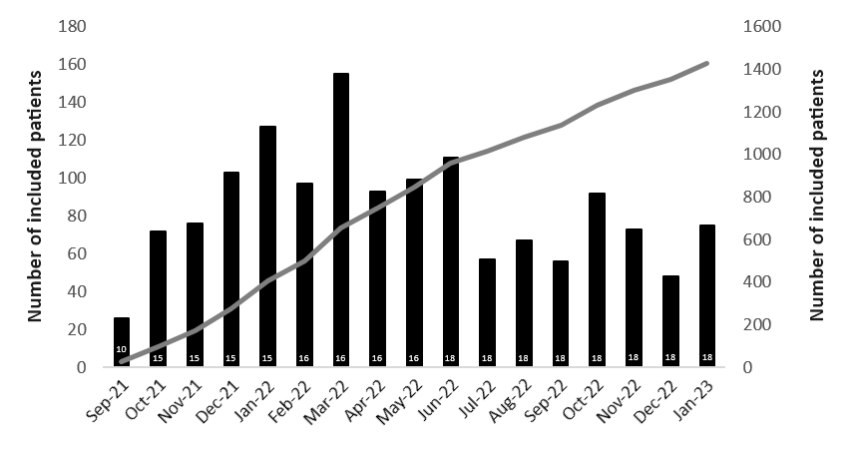
Recruitment rate by month and cumulative frequency. Rate of patient recruitment in the eDOL study is represented in terms of patient frequency/month (histogram) and cumulative frequency (curve). The numbers at the bottom of the histogram bars represent the number of active investigator centers.

All the 18 pain clinics participating in the study included at least one patient. The median rate of inclusion per center was 5.5 patients/month, and an average of 84.5 (SD 31.9) patients were consistently included every month (Figure 2). During the study period, from September 14, 2021, to January 31, 2023, 1427 patients were included.

### Collected Data

**Figure 3:**
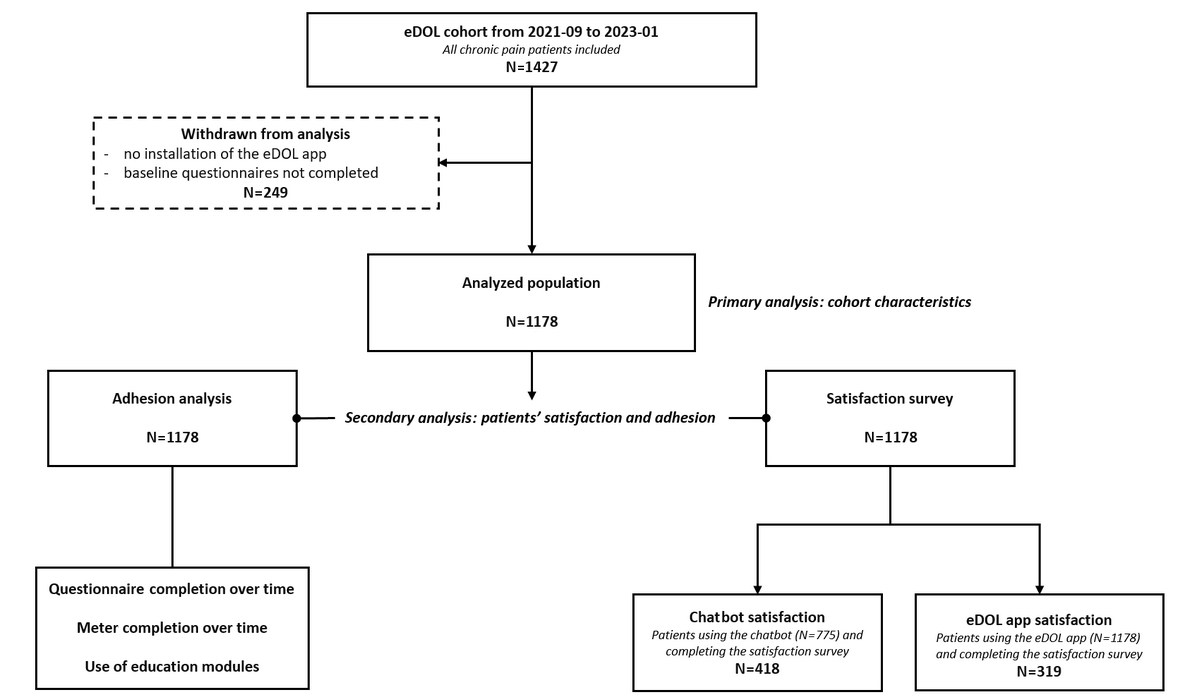
Study flowchart. Graphical representation of the various stages in patient recruitment and analysis.

Of the 1427 patients included in the study, 249 (17.4%) were not analyzable (no installation of the eDOL app or no completion of the baseline sociodemographic questionnaire). Thus, data from 1178 patients were analyzed (Figure 3). At the time of data recovery (February 1, 2023), 23.3% (275/1178) of patients had ≥12 months of follow-up, 30.1% (355/1178) had ≥9 to <12 months of follow-up, 20.5% (242/1178) had ≥6 to <9 months of follow-up, 11.5% (135/1178) had ≥3 to <6 months of follow-up, and 14.3% (168/1178) had <3 months of follow-up. The median follow-up time for patients in the cohort was 9.3 (IQR 9.1-9.5) months. eDOL’s functionalities enable it to retrieve a large and varied range of biopsychosocial data (Multimedia Appendix 1). Below is a detailed overview of the sociodemographic and medical data collected, which allowed us to precisely characterize our cohort population.

At baseline, participating patients were mostly middle-aged (median 49.0, IQR 48.3-49.7 years), women (937/1178, 79.5%), in a relationship (759/1178, 64.4%), with children (635/1178, 53.9%), nonsmoking (805/1096, 73.4%), with a university degree (581/1178, 49.3%), and in professional activity (732/1178, 62.1%), and a significant number were considered to be in a precarious state (468/980, 47.8%). Among patients in professional activity, 34.1% (250/732) were on sick leave due to their chronic pain (Multimedia Appendix 2 and Multimedia Appendix 3).

Most patients (906/1034, 87.6%) had moderate to severe pain (>5/10), of which 26.0% (236/906) had a high chronic pain interference score (called “high impact chronic pain” [45]). Moreover, 25.1% (296/1178) had at least two different types of chronic pain. Most patients (492/1178, 41.8%) had nociplastic pain, and the duration was longer than 5 years in more than 50% (601/1178, 51.0%) of patients. Most participants (667/1178, 56.6%) were at the beginning of their course in the pain clinic (≤6 months), with a median of 5.0 (IQR 3.0-7.0) months prior to inclusion.

Several patients (481/1094, 44.0%) described their chronic pain as permanent during both day and night (with painful paroxysms every day) and as inducing frequent nocturnal awakenings (995/1094, 90.9%) (Multimedia Appendix 3 and Multimedia Appendix 4). Moreover, body schemes were recovered for 73.1% (861/1178) of patients. Among these patients, 94.0% (809/861) had at least two different pain locations. The dorsolumbar, hypogastric, and cervical regions were the most frequent in terms of pain location, with over 40.0% (344/861) of patients affected (Multimedia Appendix 5).

The eDOL tool also enabled us to collect data on comorbidities related to chronic pain and its psychological and overall impact on patients’ quality of life. High numbers of patients were considered to have kinesiophobia (753/1029, 73.2%), alexithymia (591/1038, 56.9%), degraded quality of life (599/1025, 58.5%), catastrophism (448/1056, 42.4%), and a possible cognitive disorder (774/1008, 76.8%). More than 60% (640/1033, 62.0%) of patients had impaired sleep, and 44.7% (457/1023) and 26.8% (274/1023) had proven anxiety and depressive disorders, respectively (Multimedia Appendix 3 and Multimedia Appendix 6).

With regard to analgesic treatments, 2643 treatments were prescribed for 839 patients (71.2%), with 64.8% (763/1178) of patients using nondrug analgesic treatments, including transdermal electroneurostimulation (480/763, 62.9%) and physical techniques such as physiotherapy (352/763, 41.6%) (Multimedia Appendix 3 and Multimedia Appendix 7). Interestingly, 23.3% (275/1178) of patients were not receiving treatment (pharmacological or nonpharmacological) for their pain at the time of inclusion. The drugs used by the patients were mainly antidepressants (369/839, 44.0%), followed by paracetamol (363/839, 43.2%), opioids (358/839, 42.7%), and antiepileptics (224/839, 26.7%). The therapeutic benefit was assessed by clinicians for 1407 prescribed treatments, and opioids and antidepressants had the best medical benefit (1282/1407, 91.1% of treatments were rated as very good or good) (Multimedia Appendix 8). In the same way, clinicians were able to report on treatment adverse events (AEs) and patient compliance. A total of 289 AEs were reported, and antidepressants and anticonvulsants were the most likely causes (73/289, 25.4% and 66/289, 23.0% of AEs, respectively) (Multimedia Appendix 8). Compliance was assessed by clinicians for 1192 prescribed treatments, and in 97.1% (1158/1192) of cases, compliance was very good or good.

Lastly, the eDOL tool enabled clinicians to record patients’ medical history. A large majority of patients (981/1178, 83.3%) had at least one medical history (Multimedia Appendix 9). Among these patients, 60.9% (597/981) declared a traumatic life history (mainly death, accident, and divorce), 35.3% (346/981) reported a history of violence (mainly physical and psychological), 43.3% (425/981) reported a psychiatric history (mainly anxiety and depression), 37.9% (372/981) reported addictive behaviors (mainly tobacco), and 82.2% (806/981) reported ongoing illnesses (mainly rheumatologic, digestive, and neurological).

### Real-Life and Longitudinal Data Collected

As previously reported, 82.6% (1178/1427) of included patients effectively used the eDOL tool in their daily life. On average, each questionnaire was completed by 86.7% (SD 2.0%) of participants at inclusion. Among the questionnaires repeated every 3 (2 questionnaires) or 6 months (7 questionnaires), this percentage decreased over time to 60.8% (SD 0.1%) at the 3-month follow-up, 42.8% (SD 3.6%) at the 6-month follow-up, 32.1% (SD 0.1%) at the 9-month follow-up, and 23.6% (SD 2.5%) at the 12-month follow-up (Multimedia Appendix 10). 

The completion rate of the weekly assessments for the real-life monitoring of the different meters (pain, moral, anxiety, fatigue, sleep, bodily comfort, and physical activity) was 75.1% (885/1178) at patient inclusion. Among the 1007 patients with at least 3 months of follow-up, the completion rate decreased over the course of follow-up to 49.4% (497/1007) after 3 months of follow-up and then stabilized at 39.3% (108/275) for patients with at least 12 months of follow-up (Multimedia Appendix 10). An acceptable meter completion rate was defined as at least 50% completion of all theoretical meters over the follow-up period (1 meter/week). Univariate analysis was performed to characterize patients according to their percentage of weekly meter completion (<5%, ≥5% to <50%, and ≥50%). Univariate analysis showed very little difference between compliant and noncompliant patients. In fact, women and patients with an intermediate profession or no activity appeared to be slightly more compliant. Age, level of education, family situation, and alcohol or tobacco consumption had no impact on compliance. Surprisingly, the same observation was noted for pain characteristics. It was found that the length of time the pain had been present, the frequency or duration of painful paroxysms, the pain intensity and interference, and the presence of nocturnal awakenings or difficulty in falling asleep did not interact with patient compliance. There was no difference in the inclusion scores for the various questionnaires, depending on the level of compliance of patients. Finally, analysis of the type of pain and treatments received showed no difference between patients irrespective of their level of compliance.

The eDOL tool also includes educational modules on chronic pain and its repercussions. These modules were freely available to the patients but were not compulsory. Overall, 16.3% (192/1178) of patients viewed at least one educational module. The educational modules were viewed in their entirety by around 65.2% (minimum: 44.6%, maximum: 85.5%, according to the educational module) of patients who opened them. The most frequently viewed educational module was the one on “improving fatigue and sleep.” Conversely, the educational module on “living with pain” was the least viewed (Table 1).

**Table 1:** Educational module use.

Educational module	Patients opening the module (N=1178), n (%)	Patients viewing the entire module (N=1178), n (%)
Understanding pain	188 (16.0)	118 (10.0)
Knowing about treatments	181 (15.4)	129 (10.9)
Moving better	173 (14.7)	148 (12.6)
Improving fatigue and sleep	369 (31.3)	272 (23.1)
Living with pain	159 (13.5)	71 (6.0)
Managing emotions	209 (17.7)	111 (9.4)

### Satisfaction With the Use of the eDOL Tool

The satisfaction questionnaire was filled in by 27.1% (319/1178) of patients at the end of the study. These patients had similar characteristics to all the patients in the cohort (average age 49.4 years; 258/319, 80.1% female; 213/319, 66.9% with pain for more than 5 years; and 137/319, 42.8% with nociplastic pain). The median acceptability score was 7.2/10 (95% CI 6.9-7.7), with only 7.2% (23/319) of patients providing a rating less than 5/10. Moreover, 83.4% (267/319) of patients who responded wanted to participate in the further development of the eDOL tool. The items with the lowest scores were “I enjoy my exchanges with the Eva chatbot and I think it’s a tool that can assist me on a daily basis” (mean 6.0, SD 3.1), “I think the Eva chatbot is a relevant tool for collecting my meters every week” (mean 6.7, SD 2.8), and “I believe that the information I have entered in eDOL enables my doctor to better understand my pain and improve its management” (mean 6.9, SD 2.6) (Multimedia Appendix 11).

**Figure 4:**
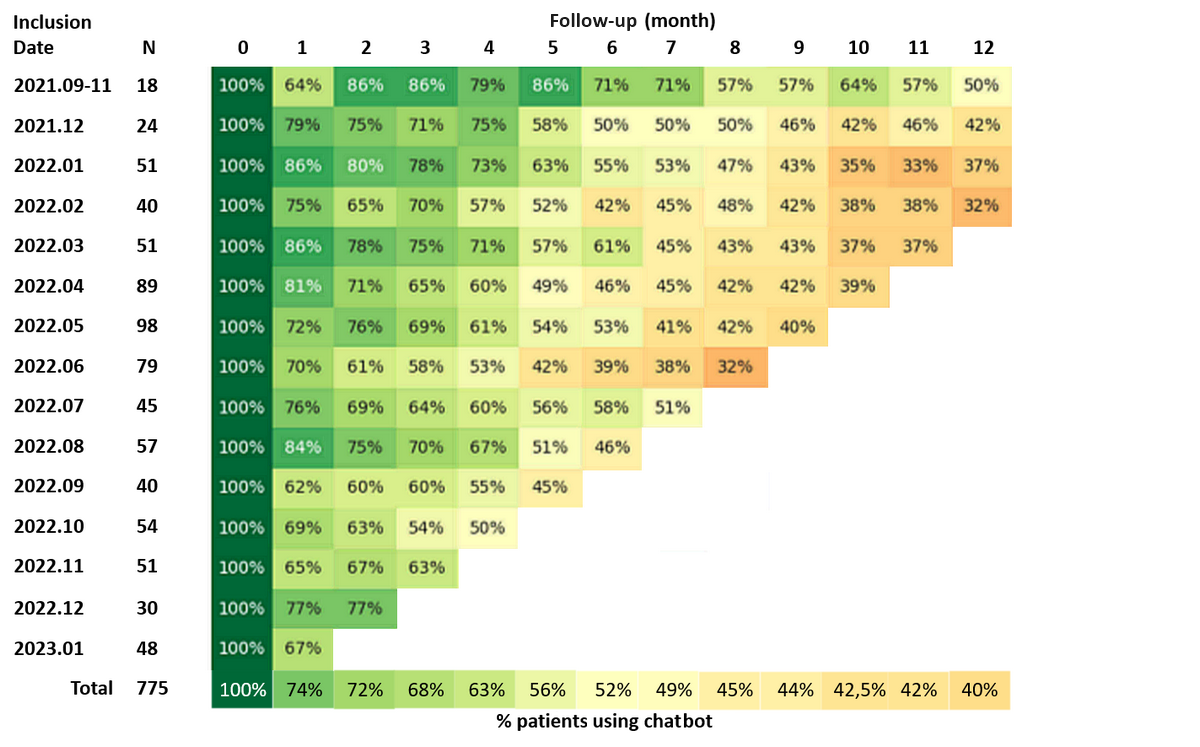
Chatbot use over time. Visual representation of chatbot use among the 775 patients who downloaded the chatbot. Patients were classified according to their month of inclusion, and chatbot use (at least one conversation in the month) was assessed for each month of follow-up. A darker green color represents a higher utilization rate, and a darker red color represents a lower utilization rate.

The interest of adding a chatbot in the eDOL tool was also assessed. Among the 1178 participating patients, 775 (65.8%) downloaded the chatbot, and among these, all 775 (100%) used the chatbot at inclusion. This percentage halved (403/775, 52.0%) at the 6-month follow-up and appeared to stabilize at 40.0% (310/775) at the 12-month follow-up (Figure 4). The use of the chatbot allowed us to recover 9456 weekly meters and 12,300 conversations. With regard to satisfaction, of the 775 patients, 418 (53.9%) responded to the questionnaire, and among these patients, 230 (55.0%) had a satisfaction score of at least 7/10, which corresponds to high satisfaction, and only 35 (8.4%) had a score below 5/10, which corresponds to low satisfaction. The main positive points of the chatbot indicated by the participants were “easy to use,” “responsive,” “moral support,” “converse with a positive message,” and “availability.” Conversely, the main difficulties related to the use of the chatbot indicated by the participants were “forgetfulness of use,” “answers sometimes not adapted,” and “repetitive.”

## Discussion

### Principal Findings

This study presents the most extensive cohort of patients using an mHealth follow-up app and living with chronic pain, irrespective of the type of pain, worldwide. Indeed, studies in the literature on the use of an mHealth app in a cohort of patients have mainly focused on specific types of pain conditions, predominantly osteoarthritis and chronic low back pain [34]. Most studies have concentrated on digital interventions directed toward the patient-provider interface [46], which makes comparisons with the available literature challenging. This is also due to the typically shorter duration of such studies.

In this cohort study, 82.6% (1178/1427) of eligible patients consented to use the eDOL tool, and among them, about 86.7% (1021/1178) of participants used the eDOL app to complete different questionnaires during the initial phase. Thus, the rate is in line with the rate identified in our previous feasibility study (89.3%) [38] and the rate reported in the study by Stoppok et al [47] wherein 85.5% of participants expressed a willingness to use the app for more than a month when surveyed beforehand. Nevertheless, attrition appears to be high in internet interventions for chronic pain [48], and this has been reflected in our study, with about 60% (167/275, 60.7%) loss of users (according to weekly meter filling) among patients with at least 12 months of follow-up. A comprehensive American study [49] involving a large sample revealed that over half of the participants discontinued participation within the first week. Attrition is not solely confined to mHealth, and a systematic review of longitudinal studies on chronic spinal pain identified attrition as the most significant risk of bias [50]. Notably, attrition rates varied widely across these longitudinal studies, with a rate of 63% after 12 months in a study assessing pain after intensive care unit discharge [51] and 83% after 12 months in a study assessing pain self-management (chronic musculoskeletal pain and comorbid depression) [52]. Moreover, in the longitudinal study conducted by Bicego et al [53], attrition rates varied within the range of 11% to 22% 1 year after treatment.

With the loss of patient adherence over time, it is necessary to include a huge number of patients (inclusion of a minimum of 5000 patients is planned in the eDOL cohort) to have enough longitudinal data available. Despite this, the eDOL tool is already making it possible to collect a significant volume of data to characterize patients experiencing chronic pain, and it opens the door to future clinical studies to better understand the biopsychosocial aspect of chronic pain, the therapeutic management (drug or nondrug) implemented, the patient’s care pathway (type of consultation, frequency of consultation, medical wandering, etc), and the cost of management (treatment prescriptions, consultations, work stoppage, etc). All these possibilities make the eDOL cohort an enormous potential source of data on chronic pain that is available to clinicians and researchers.

### Limitations and Difficulties Encountered

The main limitation of our study was the attrition rate described previously. This attrition could influence the results of patient satisfaction with the eDOL tool, as only 30% responded to the satisfaction questionnaire. However, it is difficult to know whether the patients who did not respond were dissatisfied, or simply forgot or did not feel like answering the questionnaire despite their effective satisfaction, especially since compliant patients and those who responded to the satisfaction questionnaire had similar characteristics to all the patients in the cohort. In our study, the chatbot was an attempt to add a social role to this tool in order to establish a good therapeutic alliance, as recommended for promoting engagement and reducing attrition in digital interventions [54]. However, its algorithms were trained in the United States, which might make it impersonal and irrelevant for the French population, which are characteristics identified as barriers to the use of such tools [55]. The lack of use of the chatbot could also be explained by patients’ lack of interest in “chatting” with a robot, as such a robot has shown several limitations in its exchanges. One solution would be to replace it with a chat or message involving health care staff, who have already shown an interest in adhering to the digital tool [56]. However, this strategy inevitably requires additional time for caregivers to respond to patients’ expectations, and such time is rarely available in view of caregivers’ current workloads in pain clinics. An additional possibility is to add a forum for patient users to exchange information with each other. This strategy has been proven to be beneficial [31].

Educational modules have been integrated into the eDOL app to make it more informative for patients and thus increase their adherence. Nonetheless, patients made very little use of them. Their low use can be partially explained by the fact that the modules were not available on the homepage but were present on a dedicated page of the menu. Another explanation is that patients only received 2 smartphone notifications informing them of the existence of these modules in the app. We can hypothesize that patients might have had difficulties in finding them.

Additionally, in the absence of compulsory follow-up visits in the eDOL protocol, patient visits to the pain clinics were those scheduled as part of their usual care pathway (ie, not focused on the app itself); thus, some patients had visits 3, 6, or even 12 months apart. This may have acted as a hindrance to user engagement, as noted in telemedicine interventions [57]. Furthermore, although the use of an app and a mobile phone appears to be widely preferred over other interface options to access pain-related resources among individuals living with chronic pain, less than 60% of them expressed a desire to use online pain management tools permanently, according to a German survey [47]. This finding could also explain the significant attrition commonly observed in mHealth interventions.

Various strategies are available to mitigate this adherence issue observed within this study, including reducing the number of questionnaires and the number of questions per questionnaire, simplifying navigation (user experience/user interface design), adding activities to help patients manage their symptoms (therapeutic education for sleep, mindfulness for stress, positive psychology for morale, etc), ensuring greater involvement of the care team, etc. Our research team has already taken steps to mitigate attrition rates in the ongoing novel version of the eDOL tool (Version 3; since November 2023) by reducing the number of questionnaires, replacing some questionnaires with shorter ones (PROMIS [Patient-Reported Outcomes Measurement Information System] questionnaires), enhancing user experience by designing a new interface, and deactivating the chatbot. Additionally, the reminders to complete meters, body schemes, or questionnaires were changed into notifications instead of emails as patients did not solely rely on their phones to read their emails. The limited interaction with educational modules suggests that there is an opportunity to enhance user engagement and accessibility features within the app. The new design should facilitate increased engagement with therapeutic education activities by making them more prominently visible. Additional activities are currently being prepared and will be incorporated into the new version soon. Finally, to limit missing medical data, the new version of eDOL enables us to link our data with the national databases of the French health insurance system. This linkage will make it possible to obtain data on all the care received by patients in our cohort, including treatment reimbursements, doses and quantities of treatments prescribed, hospitalizations or consultations, sick leave benefits, etc. This linkage will also enable us to carry out medicoeconomic studies on the cost of chronic pain management.

The second limitation pertains to the representativeness of the sample in this cohort regarding the general population living with chronic pain. The participants in this study were recruited exclusively from pain clinics; however, only 3% of people living with chronic pain benefit from this type of health care setting in France. The vast majority seek assistance in primary care settings or in other specialized services such as rheumatology, neurology, and oncology. Nonetheless, the characteristics of the patients included in the eDOL cohort demonstrate resemblances to people living with chronic pain in Europe in terms of various factors such as age, pain intensity, duration of pain, and comorbidities [2,58-60]. These data reveal a higher proportion of women, which is not surprising as this difference in prevalence is well documented in the literature [61-63]. This female-to-male ratio is also observed in remote research studies utilizing mobile apps (median 56.9%) [49]. However, as in our feasibility study [38], this ratio is exacerbated in our cohort, reaching nearly 8/2. There are other examples of digital health studies showing a similar gender distribution [64-66].

A selection bias was evident, primarily due to the prerequisite of smartphone utilization, which inherently excludes individuals who lack access to or proficiency with this technology. This criterion could potentially result in the exclusion of older patients or patients with a more precarious status. Lastly, another limitation could be measurement bias, a phenomenon commonly encountered in observational studies [67]. It can emerge from factors such as recall period, selective recall, social desirability, or approach to sampling. Within our study, the recall period emerged as the primary potential source of risk. Given that all the inquiries focused on the present moment or, at the most, events occurring within the preceding 1 to 2 weeks, the likelihood of recall bias can be considered marginal.

### Conclusion

Overall, the eDOL tool collected a large volume of medical and biopsychosocial data from 1178 patients over a short period of just over 1 year. The quantity of data collected demonstrated the effectiveness and interest of the eDOL tool with regard to the collection of medical data for research and for caregivers. This interest will continue to grow as more patients are included in the cohort, enabling clinical studies to be carried out on eDOL data to assess care pathways, management costs, patient trajectories, patient profiles, benefits or risks of treatments, and other aspects.

## Appendix

Multimedia Appendix 1 eDOL tool contents. Listing of all modules and functions integrated into the smartphone app and eDOL web interface.

Multimedia Appendix 2 Sociodemographic characteristics of the cohort. Graphical representation of the characteristics of patients included in the eDOL cohort. Sex, age class, marital status, alcohol or tobacco consumption, and professional status are represented in percentage of patients.

Multimedia Appendix 3 Supplementary data. All questionnaires and responses on characteristics of patients, chronic pain, and pain medications; and on biopsychosocial items related to chronic pain.

Multimedia Appendix 4 Characteristics of chronic pain–related comorbidities.

Multimedia Appendix 5 Distribution of pain locations.

Multimedia Appendix 6 Characteristics of chronic pain and daily impact. Graphical representation of the characteristics of chronic pain and its daily impact defined by the assessment of pain duration, frequency, presence of paroxysms, impact on sleep, and type of chronic pain among all included patients.

Multimedia Appendix 7 Characteristics of pain medications. Graphical representation of the characteristics of pain medications (medicines and nonmedicines) according to the percentage of patients treated among all included patients.

Multimedia Appendix 8 Medical benefits and adverse events of pain medications.

Multimedia Appendix 9 Characteristics of medical and lifestyle history. Graphical representation of the distribution of medical and lifestyle history among all included patients.

Multimedia Appendix 10 Adherence to the eDOL tool. Graphical representation of patients’ adherence to the eDOL tool reflected in the completion of their questionnaires (A) and weekly meters (B) at inclusion and at 3, 6, 9, and 12 months of follow-up.

Multimedia Appendix 11 Acceptability questionnaire data. French and English versions of the acceptability questionnaire used to assess patient satisfaction.

## Abbreviations

AE: adverse event
LIMOS: Laboratory of Informatics, Modelling and Optimization of the Systems

## References

[1] van Hecke O, Torrance N, Smith BH (2013). Chronic pain epidemiology and its clinical relevance. Br J Anaesth.

[2] Chenaf C, Delorme J, Delage N, Ardid D, Eschalier A, Authier N (2018). Prevalence of chronic pain with or without neuropathic characteristics in France using the capture-recapture method: a population-based study. Pain.

[3] GBD 2016 Disease and Injury Incidence and Prevalence Collaborators (2017). Global, regional, and national incidence, prevalence, and years lived with disability for 328 diseases and injuries for 195 countries, 1990-2016: a systematic analysis for the Global Burden of Disease Study 2016. Lancet.

[4] Breivik H, Collett B, Ventafridda V, Cohen R, Gallacher D (2012). Survey of chronic pain in Europe: Prevalence, impact on daily life, and treatment. European Journal of Pain.

[5] Attal N, Lanteri-Minet M, Laurent B, Fermanian J, Bouhassira D (2011). The specific disease burden of neuropathic pain: results of a French nationwide survey. Pain.

[6] (2017). Societal impact of pain costs the EU up to 441 billion euros annually. The Societal Impact of Pain (SIP).

[7] Gaskin DJ, Richard P (2012). The economic costs of pain in the United States. J Pain.

[8] Breivik H, Eisenberg E, O'Brien T, OPENMinds (2013). The individual and societal burden of chronic pain in Europe: the case for strategic prioritisation and action to improve knowledge and availability of appropriate care. BMC Public Health.

[9] (2021). Global medicine spending and usage trends: outlook to 2025. IQVIA.

[10] Crofford LJ (2010). Adverse effects of chronic opioid therapy for chronic musculoskeletal pain. Nat Rev Rheumatol.

[11] Mogil JS (2009). Animal models of pain: progress and challenges. Nat Rev Neurosci.

[12] Heron KE, Smyth JM (2010). Ecological momentary interventions: incorporating mobile technology into psychosocial and health behaviour treatments. Br J Health Psychol.

[13] Jamison RN, Jurcik DC, Edwards RR, Huang C, Ross EL (2017). A Pilot Comparison of a Smartphone App With or Without 2-Way Messaging Among Chronic Pain Patients: Who Benefits From a Pain App?. Clin J Pain.

[14] Dear BF, Gandy M, Karin E, Staples LG, Johnston L, Fogliati VJ, Wootton BM, Terides MD, Kayrouz R, Perry KN, Sharpe L, Nicholas MK, Titov N (2015). The Pain Course: a randomised controlled trial examining an internet-delivered pain management program when provided with different levels of clinician support. Pain.

[15] Suso-Ribera C, Castilla D, Zaragozá Irene, Ribera-Canudas MV, Botella C, García-Palacios Azucena (2018). Validity, reliability, feasibility, and usefulness of pain monitor: a multidimensional smartphone app for daily monitoring of adults with heterogenous chronic pain. Clin J Pain.

[16] Shadd JD, Ryan BL, Maddocks HL, McKay SD, Moulin DE (2015). Neuropathic pain in a primary care electronic health record database. Eur J Pain.

[17] Minen MT, Jalloh A, Ortega E, Powers SW, Sevick MA, Lipton RB (2019). User Design and Experience Preferences in a Novel Smartphone Application for Migraine Management: A Think Aloud Study of the RELAXaHEAD Application. Pain Med.

[18] Jamison RN, Mei A, Ross EL (2018). Longitudinal trial of a smartphone pain application for chronic pain patients: predictors of compliance and satisfaction. J Telemed Telecare.

[19] Stenner P, Cross V, McCrum C, McGowan J, Defever E, Lloyd P, Poole R, Moore AP (2015). Self-management of chronic low back pain: four viewpoints from patients and healthcare providers. Health Psychol Open.

[20] Coleman MT, Newton KS (2005). Supporting self-management in patients with chronic illness. Am Fam Physician.

[21] Rodríguez Sánchez-Laulhé P, Luque-Romero LG, Barrero-García F, Biscarri-Carbonero Á, Blanquero J, Suero-Pineda A, Heredia-Rizo AM (2022). An Exercise and Educational and Self-management Program Delivered With a Smartphone App (CareHand) in Adults With Rheumatoid Arthritis of the Hands: Randomized Controlled Trial. JMIR Mhealth Uhealth.

[22] Anan T, Kajiki S, Oka H, Fujii T, Kawamata K, Mori K, Matsudaira K (2021). Effects of an artificial intelligence-assisted health program on workers with neck/shoulder pain/stiffness and low back pain: randomized controlled trial. JMIR Mhealth Uhealth.

[23] Fatoye F, Gebrye T, Fatoye C, Mbada CE, Olaoye MI, Odole AC, Dada O (2020). The clinical and cost-effectiveness of telerehabilitation for people with nonspecific chronic low back pain: randomized controlled trial. JMIR Mhealth Uhealth.

[24] Anderson K, Burford O, Emmerton L (2016). Mobile health apps to facilitate self-care: a qualitative study of user experiences. PLoS One.

[25] Gogovor A, Visca R, Auger C, Bouvrette-Leblanc L, Symeonidis I, Poissant L, Ware MA, Shir Y, Viens N, Ahmed S (2017). Informing the development of an Internet-based chronic pain self-management program. Int J Med Inform.

[26] McGuire BE, Henderson EM, McGrath PJ (2017). Translating e-pain research into patient care. Pain.

[27] Sundararaman LV, Edwards RR, Ross EL, Jamison RN (2017). Integration of mobile health technology in the treatment of chronic pain: a critical review. Reg Anesth Pain Med.

[28] Martin CL, Bakker CJ, Breth MS, Gao G, Lee K, Lee MA, Tiase VL, Tunby LJ, Wyatt TH, Janeway LM (2021). The efficacy of mobile health interventions used to manage acute or chronic pain: a systematic review. Res Nurs Health.

[29] Suso-Ribera C, Castilla D, Zaragozá Irene, Mesas Á, Server A, Medel J, García-Palacios Azucena (2020). Telemonitoring in chronic pain management using smartphone apps: a randomized controlled trial comparing usual assessment against app-based monitoring with and without clinical alarms. Int J Environ Res Public Health.

[30] Ross EL, Jamison RN, Nicholls L, Perry BM, Nolen KD (2020). Clinical integration of a smartphone app for patients with chronic pain: retrospective analysis of predictors of benefits and patient engagement between clinic visits. J Med Internet Res.

[31] Mariano TY, Wan L, Edwards RR, Lazaridou A, Ross EL, Jamison RN (2021). Online group pain management for chronic pain: preliminary results of a novel treatment approach to teletherapy. J Telemed Telecare.

[32] Solomon DH, Rudin RS (2020). Digital health technologies: opportunities and challenges in rheumatology. Nat Rev Rheumatol.

[33] Alhussein G, Hadjileontiadis L (2022). Digital Health Technologies for Long-term Self-management of Osteoporosis: Systematic Review and Meta-analysis. JMIR Mhealth Uhealth.

[34] Moreno-Ligero M, Moral-Munoz JA, Salazar A, Failde I (2023). mHealth intervention for improving pain, quality of life, and functional disability in patients with chronic pain: systematic review. JMIR Mhealth Uhealth.

[35] Gordon K, Rice H, Allcock N, Bell P, Dunbar M, Gilbert S, Wallace H (2017). Barriers to self-management of chronic pain in primary care: a qualitative focus group study. Br J Gen Pract.

[36] Currie M, Philip LJ, Roberts A (2015). Attitudes towards the use and acceptance of eHealth technologies: a case study of older adults living with chronic pain and implications for rural healthcare. BMC Health Serv Res.

[37] Alexander JC, Joshi GP (2016). Smartphone applications for chronic pain management: a critical appraisal. J Pain Res.

[38] Kerckhove N, Delage N, Cambier S, Cantagrel N, Serra E, Marcaillou F, Maindet C, Picard P, Martiné G, Deleens R, Trouvin A, Fourel L, Espagne-Dubreuilh G, Douay L, Foulon S, Dufraisse B, Gov C, Viel E, Jedryka F, Pouplin S, Lestrade C, Combe E, Perrot S, Perocheau D, De Brisson V, Vergne-Salle P, Mertens P, Pereira B, Djiberou Mahamadou AJ, Antoine V, Corteval A, Eschalier A, Dualé C, Attal N, Authier N (2022). eDOL mHealth App and Web Platform for Self-monitoring and Medical Follow-up of Patients With Chronic Pain: Observational Feasibility Study. JMIR Form Res.

[39] World Medical Association (2013). World Medical Association Declaration of Helsinki: ethical principles for medical research involving human subjects. JAMA.

[40] Article R5121-13 - Code de la santé publique. Légifrance.

[41] Article 226-13 - Code pénal. Légifrance.

[42] Thayaparan AJ, Mahdi E (2013). The Patient Satisfaction Questionnaire Short Form (PSQ-18) as an adaptable, reliable, and validated tool for use in various settings. Med Educ Online.

[43] Boß L, Lehr D, Reis D, Vis C, Riper H, Berking M, Ebert DD (2016). Reliability and Validity of Assessing User Satisfaction With Web-Based Health Interventions. J Med Internet Res.

[44] Larsen DL, Attkisson CC, Hargreaves WA, Nguyen TD (1979). Assessment of client/patient satisfaction: development of a general scale. Eval Program Plann.

[45] Von Korff M, Scher AI, Helmick C, Carter-Pokras O, Dodick DW, Goulet J, Hamill-Ruth R, LeResche L, Porter L, Tait R, Terman G, Veasley C, Mackey S (2016). United States National Pain Strategy for Population Research: Concepts, Definitions, and Pilot Data. J Pain.

[46] Valentijn PP, Tymchenko L, Jacobson T, Kromann J, Biermann CW, AlMoslemany MA, Arends RY (2022). Digital Health Interventions for Musculoskeletal Pain Conditions: Systematic Review and Meta-analysis of Randomized Controlled Trials. J Med Internet Res.

[47] Stoppok P, Frewer A, Schweda A, Geiger S, Skoda E, Müßgens D, Bingel U, Teufel M, Bäuerle A (2023). Needs and Demands for eHealth Pain Management Interventions in Chronic Pain Patients. J Pers Med.

[48] Buhrman M, Gordh T, Andersson G (2016). Internet interventions for chronic pain including headache: A systematic review. Internet Interv.

[49] Pratap A, Neto EC, Snyder P, Stepnowsky C, Elhadad N, Grant D, Mohebbi MH, Mooney S, Suver C, Wilbanks J, Mangravite L, Heagerty PJ, Areán P, Omberg L (2020). Indicators of retention in remote digital health studies: a cross-study evaluation of 100,000 participants. NPJ Digit Med.

[50] Manderlier A, de Fooz M, Patris S, Berquin A (2022). Modifiable lifestyle-related prognostic factors for the onset of chronic spinal pain: A systematic review of longitudinal studies. Ann Phys Rehabil Med.

[51] Valsø Å, Rustøen T, Småstuen M, Puntillo K, Skogstad L, Schou-Bredal I, Sunde K, Tøien K (2022). Occurrence and characteristics of pain after ICU discharge: A longitudinal study. Nurs Crit Care.

[52] Damush TM, Kroenke K, Bair MJ, Wu J, Tu W, Krebs EE, Poleshuck E (2016). Pain self-management training increases self-efficacy, self-management behaviours and pain and depression outcomes. Eur J Pain.

[53] Bicego A, Monseur J, Collinet A, Donneau A, Fontaine R, Libbrecht D, Malaise N, Nyssen A, Raaf M, Rousseaux F, Salamun I, Staquet C, Teuwis S, Tomasella M, Faymonville M, Vanhaudenhuyse A (2021). Complementary treatment comparison for chronic pain management: A randomized longitudinal study. PLoS One.

[54] Kassinopoulos O, Vasiliou V, Karekla M (2023). Overcoming challenges in adherence and engagement digital interventions: The development of the ALGEApp for chronic pain management. Internet Interv.

[55] Fernandes LG, Devan H, Fioratti I, Kamper SJ, Williams CM, Saragiotto BT (2022). At my own pace, space, and place: a systematic review of qualitative studies of enablers and barriers to telehealth interventions for people with chronic pain. Pain.

[56] Mariano TY, Wan L, Edwards RR, Jamison RN (2021). Online teletherapy for chronic pain: A systematic review. J Telemed Telecare.

[57] Cascella M, Marinangeli F, Vittori A, Scala C, Piccinini M, Braga A, Miceli L, Vellucci R (2021). Open Issues and Practical Suggestions for Telemedicine in Chronic Pain. Int J Environ Res Public Health.

[58] Azevedo LF, Costa-Pereira A, Mendonça L, Dias CC, Castro-Lopes JM (2012). Epidemiology of chronic pain: a population-based nationwide study on its prevalence, characteristics and associated disability in Portugal. J Pain.

[59] Köppen P, Dorner TE, Stein KV, Simon J, Crevenna R (2018). Health literacy, pain intensity and pain perception in patients with chronic pain. Wien Klin Wochenschr.

[60] Fayaz A, Croft P, Langford RM, Donaldson LJ, Jones GT (2016). Prevalence of chronic pain in the UK: a systematic review and meta-analysis of population studies. BMJ Open.

[61] Larsson C, Hansson EE, Sundquist K, Jakobsson U (2017). Chronic pain in older adults: prevalence, incidence, and risk factors. Scand J Rheumatol.

[62] Andrews P, Steultjens M, Riskowski J (2018). Chronic widespread pain prevalence in the general population: a systematic review. Eur J Pain.

[63] Jackson T, Thomas S, Stabile V, Han X, Shotwell M, McQueen K (2015). Prevalence of chronic pain in low-income and middle-income countries: a systematic review and meta-analysis. Lancet.

[64] Pratap A, Renn BN, Volponi J, Mooney SD, Gazzaley A, Arean PA, Anguera JA (2018). Using mobile apps to assess and treat depression in Hispanic and Latino populations: fully remote randomized clinical trial. J Med Internet Res.

[65] Anguera JA, Jordan JT, Castaneda D, Gazzaley A, Areán P (2016). Conducting a fully mobile and randomised clinical trial for depression: access, engagement and expense. BMJ Innov.

[66] Crouthamel M, Quattrocchi E, Watts S, Wang S, Berry P, Garcia-Gancedo L, Hamy V, Williams RE (2018). Using a ResearchKit Smartphone App to Collect Rheumatoid Arthritis Symptoms From Real-World Participants: Feasibility Study. JMIR Mhealth Uhealth.

[67] Althubaiti A (2016). Information bias in health research: definition, pitfalls, and adjustment methods. J Multidiscip Healthc.

