# Supplementary figures and images for: Mobile Health App and Web Platform (eDOL) for Medical Follow-Up of Patients With Chronic Pain: Cohort Study Involving the French eDOL National Cohort After 1 Year
Source: JMIR Mhealth Uhealth. 2024 Jun 12;12:e54579. doi: 10.2196/54579 (PMC11208841; doi:10.2196/54579)

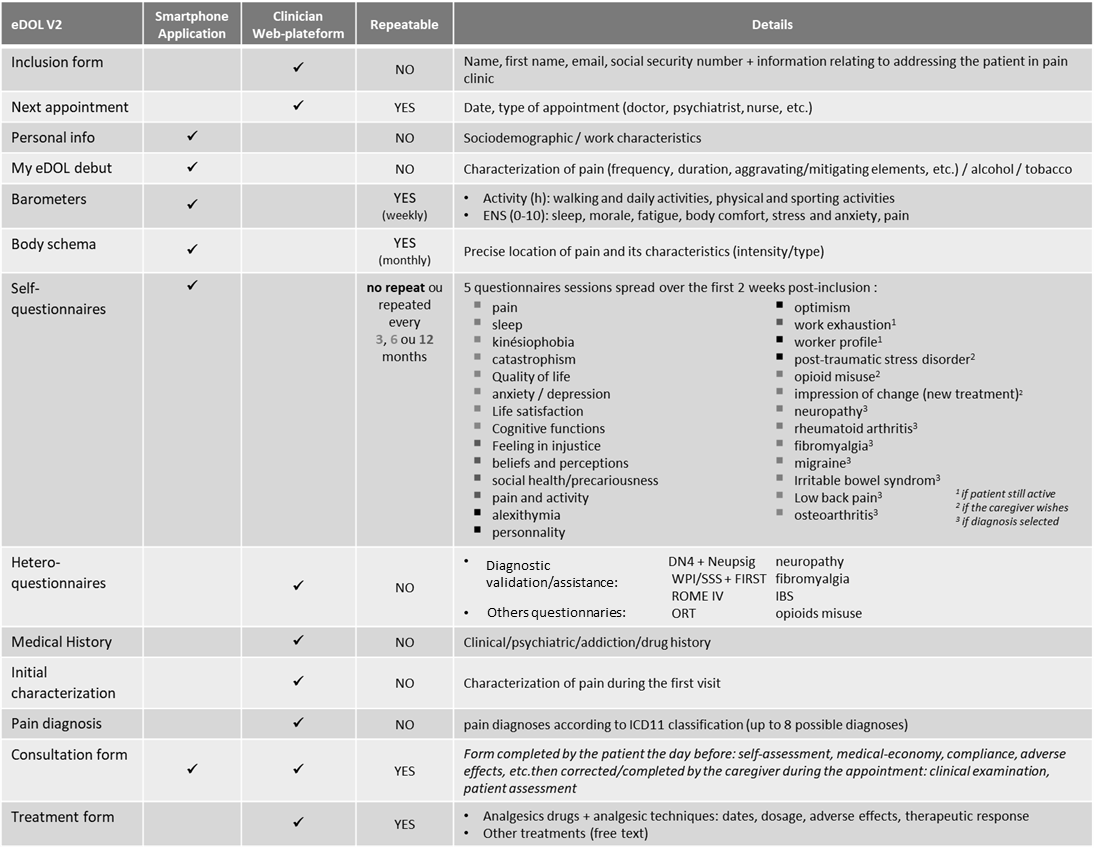

Supplement: Multimedia Appendix 1 [file mhealth_v12i1e54579_app1.png]

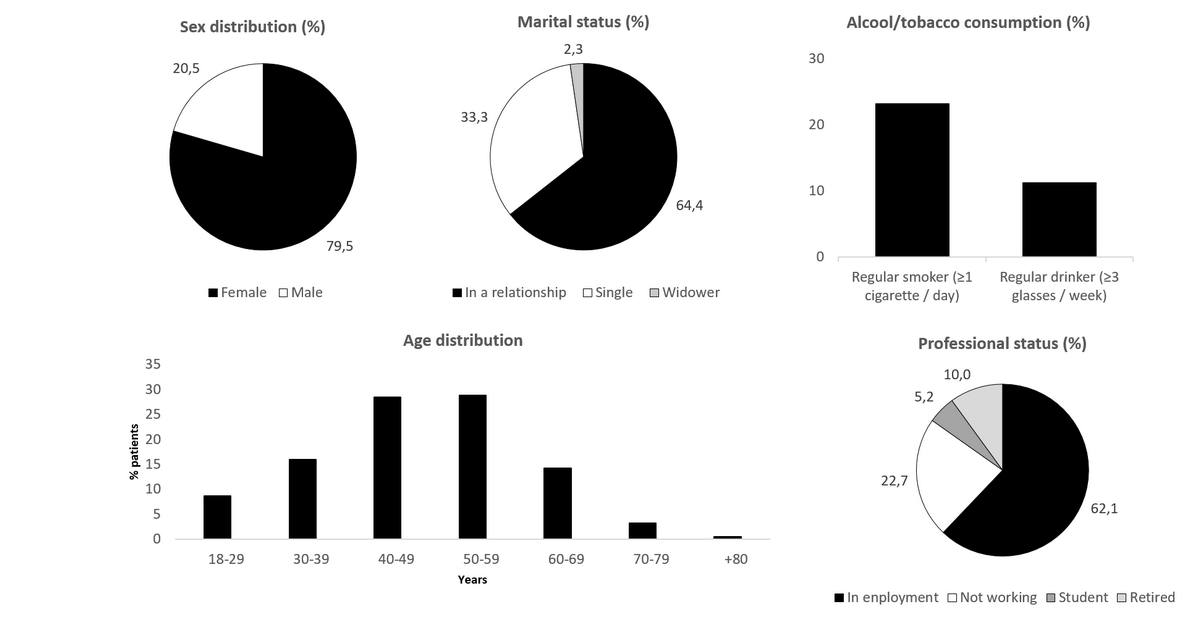

Supplement: Multimedia Appendix 2 [file mhealth_v12i1e54579_app2.png]

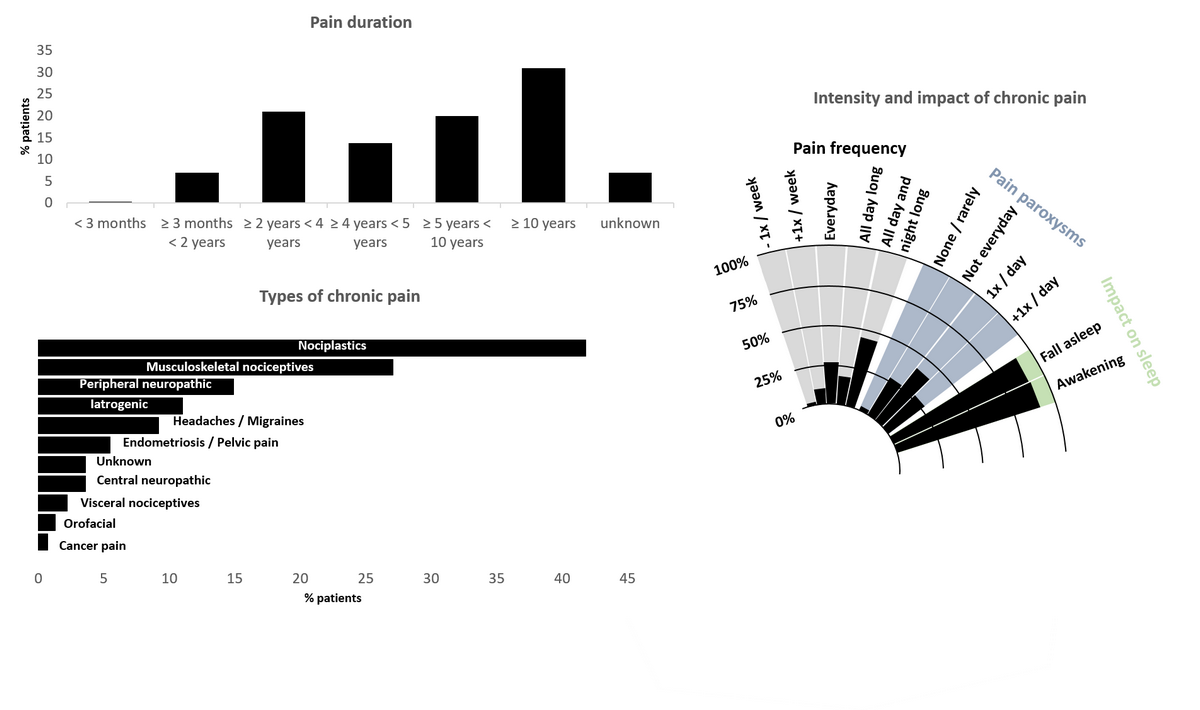

Supplement: Multimedia Appendix 6 [file mhealth_v12i1e54579_app6.png]

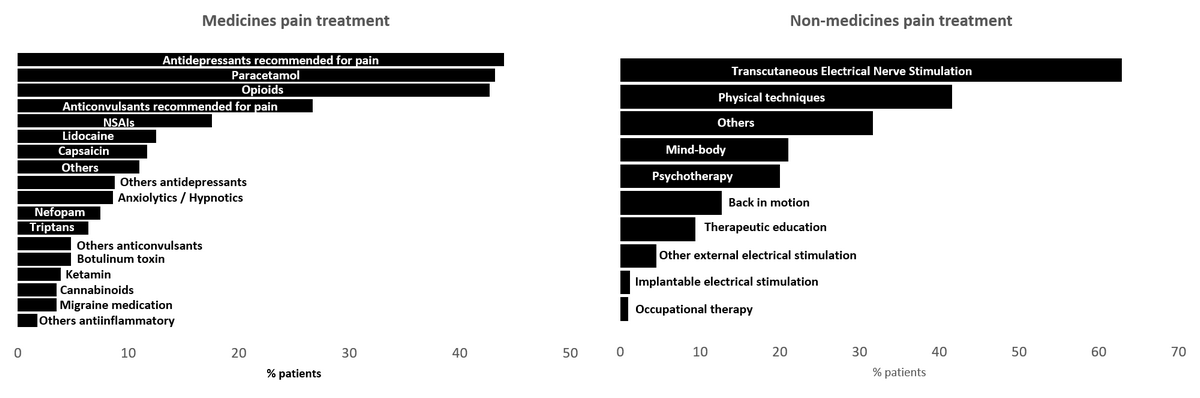

Supplement: Multimedia Appendix 7 [file mhealth_v12i1e54579_app7.png]

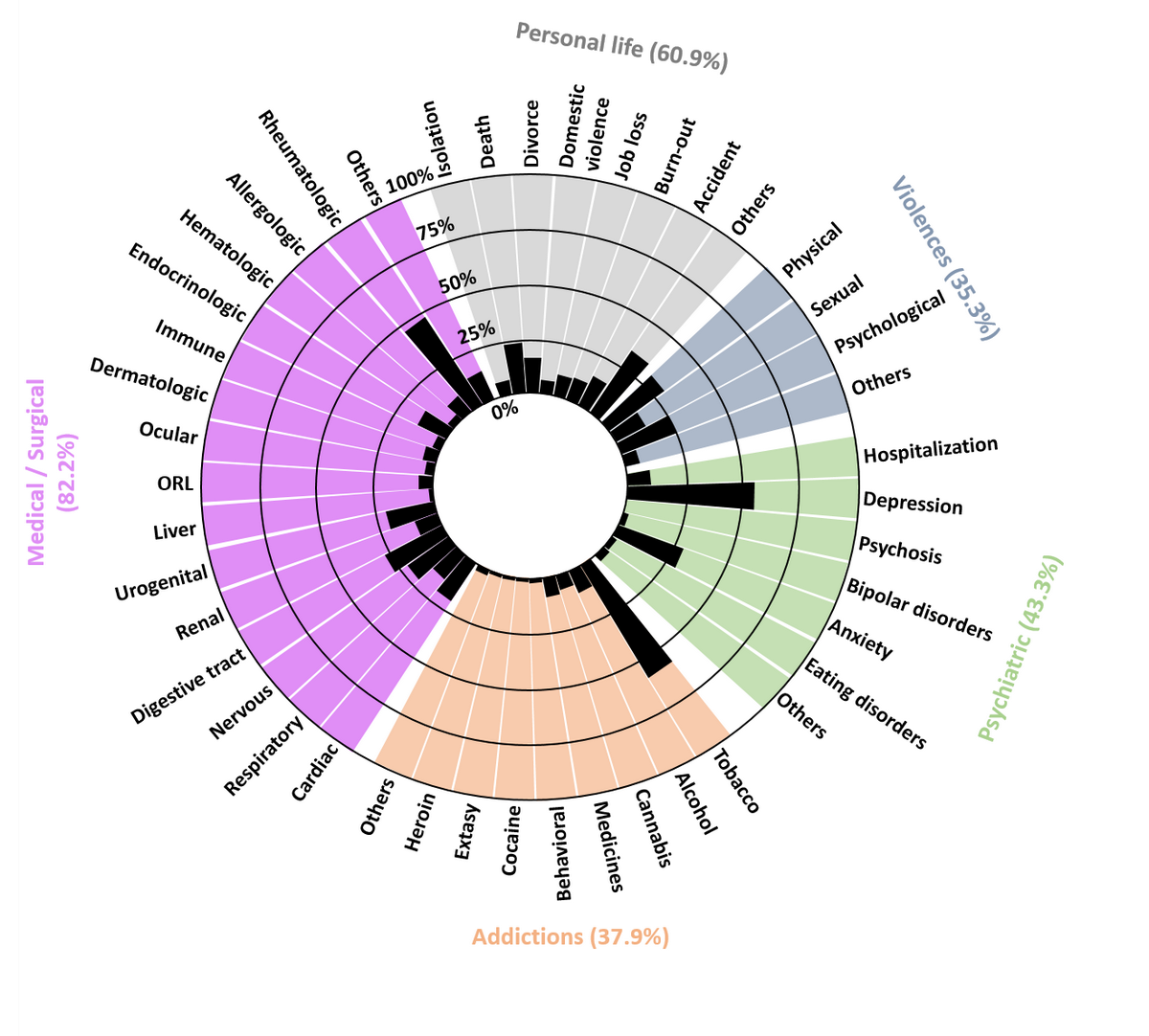

Supplement: Multimedia Appendix 9 [file mhealth_v12i1e54579_app9.png]

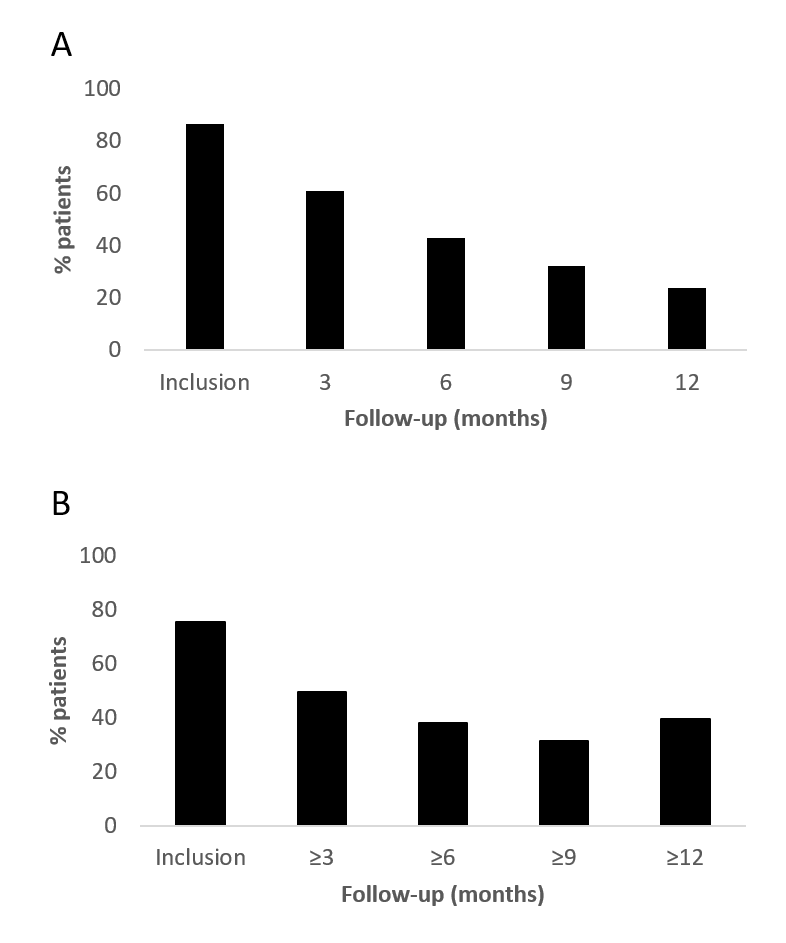

Supplement: Multimedia Appendix 10 [file mhealth_v12i1e54579_app10.png]

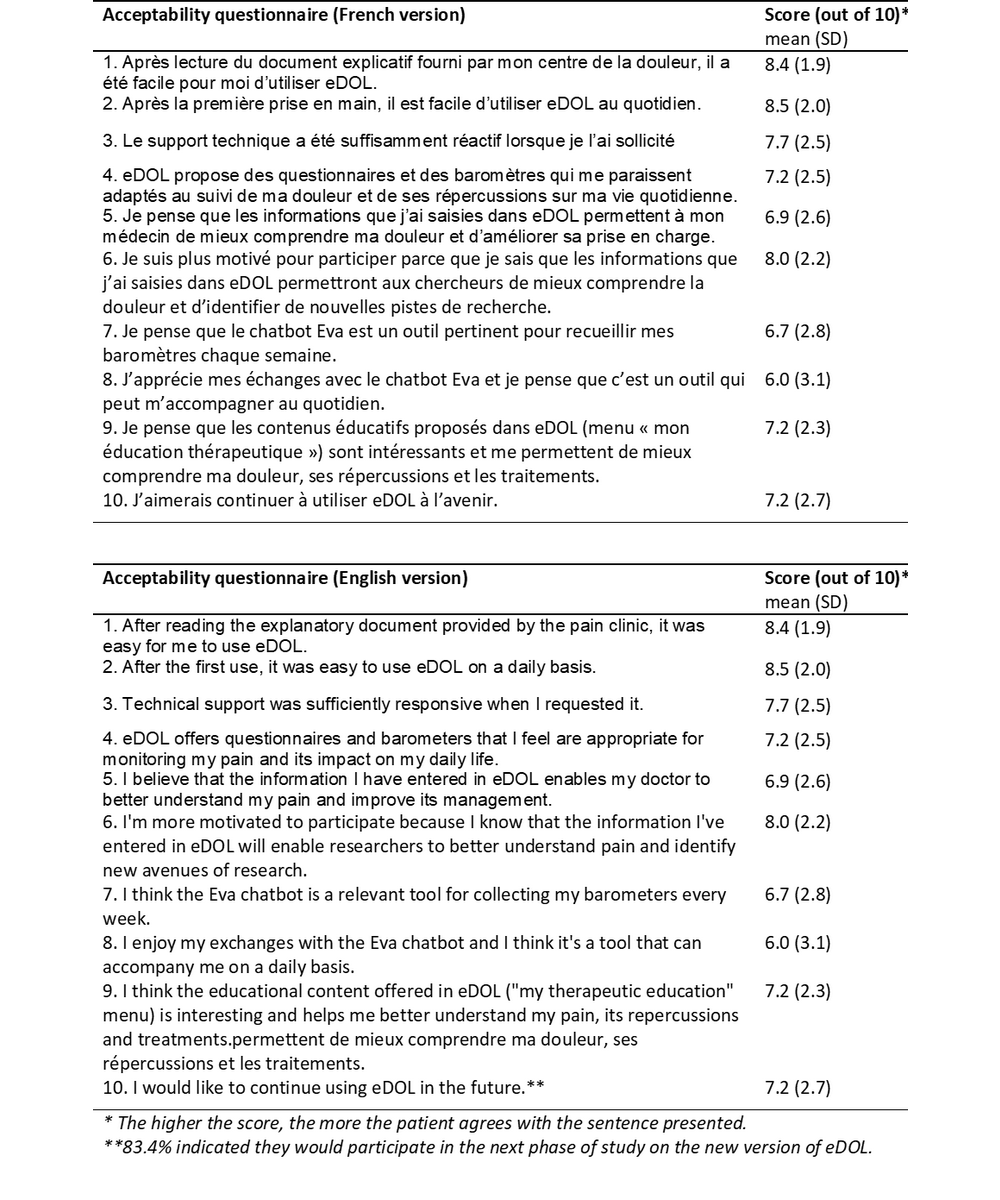

Supplement: Multimedia Appendix 11 [file mhealth_v12i1e54579_app11.png]
