# Supplementary material for: Mobile Health App and Web Platform (eDOL) for Medical Follow-Up of Patients With Chronic Pain: Cohort Study Involving the French eDOL National Cohort After 1 Year
Source: JMIR Mhealth Uhealth. 2024 Jun 12;12:e54579. doi: 10.2196/54579 (PMC11208841; doi:10.2196/54579)
Supplement: Multimedia Appendix 3 [file mhealth_v12i1e54579_app3.docx]

**Multimedia Appendix 3.** Supplementary data.

**Table S1. Questionnaires on characteristics of patients, chronic pain, and pain medications.**

| **DEMOGRAPHIC DATA (n = 1,178)** |
| --- |
| Female = 79.5% (937) |
| Male = 20.1% (237) |
| Non-Binary = 0.08% (1) |
| Missing data = 0.2% (3) |
| Age (years, median [CI95%]) = 49.0 [48.3;49.7] |
| Ages classes |
| *18-29 years = 8,7% (102)* |
| *30-39 years = 16,0% (189)* |
| *40-49 years = 28,5% (336)* |
| *50-59 years = 28,8% (340)* |
| *60-69 years = 14,2% (167)* |
| *70-79 years = 3,2% (38)* |
| *+80 years = 0,5% (6)* |
| eDOL follow-up time (months, median [CI95%], min-max) = 11.0 [10.7;11.3] (0.75-18.0) |
| In relationship = 64.4% (759) |
| Single = 33.3% (392) |
| Widower = 2.3% (27) |
| With child = 53.9% (635) |
| **Tobacco/Alcohol (n = 1,096)** |
| Non-Smoking = 73.4% (805) |
| Abstinent alcohol = 37.5% (411) |
| **Precariousness (n = 980)** |
| Assumed precariousness = 47.8% (468) |
| **Education level (n = 1,178)** |
| General certificate of secondary education = 6.4% (75) |
| NVQ = 11.4% (134) |
| BTEC / GNVQ = 11.5% (136) |
| A level = 21.4% (252) |
| Diploma of Higher Education = 17.1% (201) |
| Bachelor = 17.0% (200) |
| Master degree and beyond = 15.3% (180) |
| **Socioprofessional category (n = 1,178)** |
| Farmers = 0.7% (8) |
| Craftsmen/merchants/business owners = 3.8% (45) |
| Managers = 18.1% (213) |
| Employees = 38.1% (449) |
| Manual workers = 4.0% (47) |
| Intermediate professions = 8.1% (95) |
| Without vocational training = 27.2% (321) |
| **Professional status (n = 1,178)** |
| In professional activity = 62.1% (732) |
| *Off work due to chronic pain = 34.1% (250/732)* |
| *Off work not related to chronic pain = 3.7% (27/732)* |
| *Disabled Adult Allowance = 1.2% (9/732)* |
| *Invalidity = 6.6% (48/732)* |
| *Earned income supplement = 0.5% (4/732)* |
| No professional activity = 22.7% (267) |
| *Unemployment = 32.6% (87/267)* |
| *Earned income supplement = 6.4% (17/267)* |
| *Invalidity = 56.6% (151/267)* |
| *Disabled Adult Allowance = 24.0% (64/267)* |
| Student = 5.2% (61) |
| Retired = 10.0% (118) |
| **CHRONIC PAIN DATA (n = 1,178)** |
| **Chronic pain duration (n = 1,178)** |
| < 3 months = 0.3% (4) |
| 3 months - 1 year = 7.0% (82) |
| 2 - 3 years = 21.0% (248) |
| 4 - 5 years = 13.7% (161) |
| >5 - 10 years = 20.0% (236) |
| >10 years = 31.0% (365) |
| Unknown = 7.0% (82) |
| Pain clinic follow-up time (months, median [CI95%], min-max) = 5,0 [3,0;7,0] (0-244) |
| **Intensity and daily impact of pain symptoms (n = 1,094)** |
| Interference with sleep |
| Difficulty falling asleep = 90.7% (992) |
| Nocturnal awakenings = 90.9% (995) |
| Frequency of pain |
| < once a week = 1.0% (11) |
| Several times a week = 9.8% (108) |
| Every day = 26.7% (292) |
| Continuous during the day = 18.5% (202) |
| Continuous day and night = 44.0% (481) |
| Frequency of painful paroxysms |
| Never or rarely = 2.2% (24) |
| Not every day = 27.3% (299) |
| Once a day = 41.9% (458) |
| Several times a day = 28.6% (313) |
| Intensity and daily interference |
| Overall intensity (score /10, mean) = 5.8±1.9 |
| Interference with daily activities (score /10, mean) = 4.0±1.3 |
| High impact chronic pain (Intensity ≥4/10 and interference ≥5/10) = 26.0% (236/906) |
| **Type of chronic pain (n = 1,178)** |
| Central neuropathic pain = 3.6% (43) |
| Peripheral neuropathic pain = 14.9% (176) |
| Musculoskeletal nociceptive pain = 27.1% (319) |
| Visceral nociceptive pain = 2.2% (26) |
| Nociplastic pain = 41.8% (492) |
| Headache / migraine = 9.2% (108) |
| Orofacial pain = 1.3% (15) |
| Endometriosis / pelvic pain = 5.5% (65) |
| Iatrogenic pain = 11.0% (130) |
| Cancer pain = 0.7% (8) |
| Unknown = 3.6% (42) |
| Patients with ≥2 types of chronic pain = 24.2% (296) |
| **PAIN MEDICATIONS DATA (n = 1,178)** |
| **Pharmacological analgesic treatments (n = 839)** |
| Paracetamol = 43.2% (363) |
| NSAIDs = 17.6% (148) |
| Other anti-inflammatories = 1.8% (15) |
| Antirheumatic = 0 (0) |
| Opioids = 42.7% (358) |
| Triptans = 6.4% (54) |
| Other antimigraine drugs = 3.5% (29) |
| Antidepressants with marketing authorization or recommended for pain = 44.0% (369) |
| Other antidepressants = 8.8% (74) |
| Antiepileptics with marketing authorization or recommended for pain = 26.7% (224) |
| Other antiepileptics = 4.8% (40) |
| Anxiolytics & hypnotics = 8.6% (72) |
| Capsaicin = 11.7% (98) |
| Ketamine = 3.9% (33) |
| Lidocain = 12.5% (105) |
| Nefopam = 7.5% (63) |
| Cannabinoids = 3.5% (29) |
| Botulinum toxin = 4.8% (40) |
| Other = 11.0% (92) |
| None = 28.8% (339) |
| **Non-pharmacological analgesic techniques (n = 763)** |
| TENS = 62.9% (480) |
| Other external stimulation = 4.5% (34) |
| Implantable stimulation = 1.2% (9) |
| Physical techniques = 41.6% (352) |
| Restoration of movement = 12.7% (97) |
| Psychotherapeutic approaches = 20.0% (153) |
| Body-psychological approaches = 21.1% (161) |
| Occupational therapy = 1.0% (8) |
| Therapeutic education = 9.4% (72) |
| Other approaches = 31.7% (242) |
| None = 35.2% (415) |
| No pharmacological and non-pharmacological treatment = 23.3% (275) |
| **MEDICAL HISTORY DATA (n = 981)** |
| Life history = 60.9% (597) |
| Social and family isolation = 7.9% (47/597) |
| Death of a loved one = 23.1% (138/597) |
| Divorce = 16.9% (101/597) |
| Domestic violence = 5.9% (35/597) |
| Job loss = 10.4% (62/597) |
| Burn-out = 11.9% (71/597) |
| Accident = 15.1% (90/597) |
| Other = 34.5% (206/597) |
| Violence = 35.3% (346) |
| Physical = 31.5% (109/346) |
| Sexual = 15.6% (54/346) |
| Psychological = 24.9% (86/346) |
| Other = 7.2% (25/346) |
| Psychiatric = 43.3% (425) |
| Psychiatric hospitalization = 10.6% (45/425) |
| Depression = 55.8% (237/425) |
| Psychosis = 0.0% (0/425) |
| Bipolar disorder = 4.0% (17/425) |
| Anxiety = 30.3% (129/425) |
| Eating disorders = 3.8% (16/425) |
| Other = 5.9% (25/425) |
| Addictive behavior = 37.9% (372) |
| Tobacco = 59.7% (222/372) |
| Alcohol = 11.0% (41/372) |
| Cannabis = 7.5% (28/372) |
| Medication = 9.9% (37/372) |
| Behavioral addictions = 1.9% (7/372) |
| Cocaine = 1.9% (7/372) |
| Ecstasy = 0.5% (2/372) |
| Heroin = 0.8% (3/372) |
| Other = 1.3% (5/372) |
| Medical/surgical = 82.2% (806) |
| Cardioc = 20.3% (164/806) |
| Respiratory = 14.8% (119/806) |
| Nervous = 22.6% (182/806) |
| Digestive tract = 28.5% (230/806) |
| Renal = 10.3% (83/806) |
| Urogenital = 21.0% (169/806) |
| Liver = 1.5% (12/806) |
| OthoRhinoLaryngology = 6.6% (53/806) |
| Ocular = 4.0% (32/806) |
| Dermatologic = 6.2% (50/806) |
| Immune = 3.7% (30/806) |
| Endocrinologic = 14.9% (120/806) |
| Hematologic = 3.0% (24/806) |
| Allergologic = 9.8% (79/806) |
| Rheumatologic = 47.4% (382/806) |
| Others = 14.9% (120/806) |

**Table S2. Questionnaires on biopsychosocial items related to chronic pain.**

|  |  | **n quest.** | **% patients (n) or**  **score questionnaires*** |
| --- | --- | --- | --- |
| **Pain Perception** | Catastrophism | 1056 | 42.4% (448) |
|  | Kinesiophobia | 1029 | 73.2% (753) |
|  | Sense of guilt (score -5 to +5) | 991 | -4.0±3.1 |
|  | Sense of mystery (score -5 to +5) | 991 | 1.0±4.0 |
|  | Sense of permanence (score -5 to +5) | 991 | 3.0±3.8 |
|  | Sense of constancy (score -5 to +5) | 991 | 1.0±2.9 |
| **Psychologic profile** | Personality inventive/curious (score / 5) | 988 | 3.5±0.7 |
|  | Personality sensitive/nervous (score / 5) | 988 | 3.4±0.8 |
|  | Personality efficient/organized (score / 5) | 988 | 4.0±0.6 |
|  | Personality friendly/compassionate (score / 5) | 988 | 4.1±0.5 |
|  | Personality outgoing/energetic (score / 5) | 988 | 3.1±0.8 |
|  | Alexithymia | 1038 | 56.9% (591) |
|  | Optimism (score /40) | 1006 | 12.0±2.1 |
|  | Belief in justice (score /30) | 1006 | 17.2±4.2 |
|  | Sense of injustice (score /48) | 1042 | 27.2±10.2 |
| **Comorbidities** | Anxiety | 1023 | 44.7% (457) |
|  | Depression | 1023 | 26.8% (274) |
|  | Cognitive disorders | 1008 | 76.8% (774) |
|  | Sleep disorders | 1025 | 62.4% (640) |
| **Quality of life** | Health status (score /100) | 1023 | 49.0±20.0 |
|  | Pain (score /3) | 1023 | 2.0±0.7 |
|  | Anxiety (score /3) | 1023 | 2.4±0.5 |
|  | Activity (score /3) | 1023 | 1.8±0.5 |
|  | Autonomy (score /3) | 1023 | 1.2±0.4 |
|  | Mobility (score /3) | 1023 | 1.5±0.5 |
|  | Impact of pain on daily life (score /66) | 1022 | 13.7±5.2 |
|  | Dissatisfaction with life | 1066 | 50.6% (539) |
| * The higher the score, the greater the disturbance/personality | | | |
