# Supplementary material for: Mobile Health App and Web Platform (eDOL) for Medical Follow-Up of Patients With Chronic Pain: Cohort Study Involving the French eDOL National Cohort After 1 Year
Source: JMIR Mhealth Uhealth. 2024 Jun 12;12:e54579. doi: 10.2196/54579 (PMC11208841; doi:10.2196/54579)
Supplement: Multimedia Appendix 4 [file mhealth_v12i1e54579_app4.docx]

**Multimedia Appendix 4.** Characteristics of chronic pain–related comorbidities.

Frequency of main comorbidities related to the presence of chronic pain. Comorbidities were assessed using validated questionnaires: Pain Catastrophizing Scale (catastrophism), Tampa Scale Kinesiophobia (kinesophobia), Toronto Alexithymia Scale-20 (alexithymia), Hospital Anxiety and Depression scale (anxiety and depression), Consciousness Perception Questionnaire (cognition), MOS Sleep Scale (sleep), and EQ-5D-3L (quality of life).

| **Items related to chronic pain** | **% patients** |
| --- | --- |
| Catastrophism | 42.4% (448/1,056) |
| Kinesiophobia | 73.2% (753/1,029) |
| Alexithymia | 56.9% (591/1,038) |
| Anxiety | 44.7% (457/1,023) |
| Depression | 26.8% (274/1,023) |
| Cognitive disorders | 76.8% (774/1,008) |
| Sleep disorders | 62.0% (640/1,033) |
| Quality of Life |  |
| *Very good* | 4.8% (49/1,024) |
| *Good* | 36.3% (372/1,024) |
| *Normal* | 38.1% (390/1,024) |
| *Bad/Very bad* | 20.8% (213/1,024) |
