# Supplementary material for: Mobile Health App and Web Platform (eDOL) for Medical Follow-Up of Patients With Chronic Pain: Cohort Study Involving the French eDOL National Cohort After 1 Year
Source: JMIR Mhealth Uhealth. 2024 Jun 12;12:e54579. doi: 10.2196/54579 (PMC11208841; doi:10.2196/54579)
Supplement: Multimedia Appendix 5 [file mhealth_v12i1e54579_app5.docx]

**Multimedia Appendix 5.** Distribution of pain locations.

Listing and distribution (%) of chronic pain locations among all patients who completed their body schemes.

| **Pain locations** | **% patients** (n=860) |
| --- | --- |
| Lumbosacral region | 46.9 |
| Back neck | 45.8 |
| Hypogastrium | 40.8 |
| Dorso-lumbar region | 40.4 |
| Front thigh left | 39.4 |
| Upper back region | 39.1 |
| Front thigh right | 38.9 |
| Back shoulder right | 38.1 |
| Chest | 38.0 |
| Knee right | 35.8 |
| Knee left | 35.7 |
| Shoulder right | 35.5 |
| Upper foot left | 35.5 |
| Upper foot right | 35.2 |
| Shoulder left | 33.9 |
| Front neck | 33.8 |
| Back shoulder left | 33.8 |
| Rear buttock right | 33.8 |
| Rear buttock left | 31.8 |
| Inside hand and fingers right | 30.9 |
| Inside hand and fingers left | 30.5 |
| Parietal region | 29.7 |
| Inside wrist right | 28.0 |
| Parietal region | 27.9 |
| Front upper arm right | 26.0 |
| Front forearm right | 25.6 |
| Inside wrist left | 25.2 |
| Front calf left | 25.1 |
| Front calf right | 24.3 |
| Inside elbow right | 23.8 |
| Front ankle left | 23.8 |
| Front upper arm left | 23.2 |
| Inside elbow left | 23.1 |
| Front ankle right | 22.8 |
| Rear thigh left | 22.6 |
| Rear thigh right | 22.5 |
| Front forearm left | 22.3 |
| Flank right | 21.0 |
| Under foot right | 20.4 |
| Under foot left | 19.9 |
| Rear arm right | 19.7 |
| Rear calf left | 19.5 |
| Back knee right | 19.3 |
| Flank left | 19.0 |
| Rear knee left | 19.0 |
| Upper hand and fingers left | 18.9 |
| Rear flank right | 18.9 |
| Upper hand and fingers right | 18.7 |
| Rear elbow right | 18.6 |
| Rear arm left | 18.4 |
| Rear calf right | 18.4 |
| Rear forearm right | 17.9 |
| Back wrist right | 17.9 |
| Rear ankle left | 17.9 |
| Back wrist left | 17.4 |
| Rear elbow left | 17.2 |
| Rear ankle right | 16.5 |
| Back forearm left | 16.1 |
| Rear flank left | 16.0 |
| Epigastrium | 15.6 |
| Mandibular region right | 14.6 |
| Sex | 14.6 |
| Mandibular region left | 13.6 |
| Orbital region | 11.5 |
| Orbital region | 10.2 |
| Chin | 10.0 |
| Anus | 6.2 |
| Ear right | 6.0 |
| Nasal region | 5.9 |
| Ear left | 5.8 |
| Mouth | 5.3 |
| Back ear right | 4.6 |
| Back ear left | 3.4 |
