# Supplementary material for: Mobile Health App and Web Platform (eDOL) for Medical Follow-Up of Patients With Chronic Pain: Cohort Study Involving the French eDOL National Cohort After 1 Year
Source: JMIR Mhealth Uhealth. 2024 Jun 12;12:e54579. doi: 10.2196/54579 (PMC11208841; doi:10.2196/54579)
Supplement: Multimedia Appendix 8 [file mhealth_v12i1e54579_app8.docx]

**Multimedia Appendix 8.** Medical benefits and adverse events of pain medications.

Assessment of the benefits and risks of drug therapies according to clinicians’ opinions.

| **Therapeutic classes / drug*** | **Medical benefits** (% patients)** | | | | **Adverse events**  **(% of patients)** |
| --- | --- | --- | --- | --- | --- |
|  | **Very good** | **Good** | **Bad** | **Very bad** |  |
| NSAIDs (n=96) | 11.5 | 78.1 | 7.3 | 3.1 | 8.3 |
| Antidepressants*** (n=260) | 16.5 | 74.6 | 7.3 | 1.5 | 25.4 |
| Anticonvulsants*** (n=183) | 14.2 | 69.4 | 16.4 | 0.0 | 23.0 |
| Opioids (n=286) | 19.9 | 72.0 | 7.7 | 0.4 | 19.2 |
| Paracetamol (n=108) | 11.1 | 63.0 | 25.0 | 0.9 | 0.9 |
| ** The number in brackets corresponds to the number of drugs assessed by clinicians*  *** According to clinicians’ assessment*  **** Recommended for chronic pain* | | | | | |
